# Influence of the adhesive strategy in the sealing ability of resin composite inlays after deep margin elevation

**DOI:** 10.4317/jced.58689

**Published:** 2021-09-01

**Authors:** Dayana Da Silva, Laura Ceballos, María-Victoria Fuentes

**Affiliations:** 1 DDS, PhD, Assistant Professor, IDIBO Research Group, Nursing and Stomatology Department, Health Sciences Faculty, Rey Juan Carlos University. Av. de Atenas, S/N, 28922. Alcorcón, Madrid, Spain; 2 DDS, PhD, Professor, IDIBO Research Group, Nursing and Stomatology Department, Health Sciences Faculty, Rey Juan Carlos University. Av. de Atenas, S/N, 28922. Alcorcón, Madrid, Spain; 3 DDS, PhD, Associate Professor, IDIBO Research Group, Nursing and Stomatology Department, Health Sciences Faculty, Rey Juan Carlos University. Av. de Atenas, S/N, 28922. Alcorcón, Madrid, Spain

## Abstract

**Background:**

The aim of this study was to determine the influence of the gingival margin position and the adhesive strategy selected to perform deep margin elevation (DME) in marginal sealing of resin composite inlays by a nanoleakage test.

**Material and Methods:**

12 sound third molars were selected and expulsive MOD cavities for inlays were prepared. Experimental groups were established according to gingival margin location (enamel: 1 mm above cemento-enamel junction (CEJ), dentin: 1 mm below CEJ, or DME, and the adhesive strategy used to lute inlays and elevate the gingival margin. Therefore, the six experimental groups were: 1) Enamel + etch-and-rinse adhesive (ERA) Adper Scotchbond 1XT (SB1XT); 2) Dentin + SB1XT; 3) DME + SB1XT; 4) Enamel + self-etching adhesive (SEA) with enamel selective etching Clearfil SE Bond (CSE); 5) Dentin + CSE; 6) DME + CSE. Resin composite inlays were constructed (Gradia Indirect) and all luted with the same resin cement (RelyX ARC). Specimens were submitted to nanoleakage test. Results were analyzed by Kruskal-Wallis and Mann-Whitney U tests with Bonferroni correction (*p*<0.05).

**Results:**

A perfect sealing ability was evidenced for experimental groups with gingival margins on enamel. Similar nanoleakage values were determined when CSE adhesive was applied regardless the gingival margin position. The highest silver nitrate infiltration was detected for elevated margins with the ERA SB1XT.

**Conclusions:**

The SEA Clearfil SE Bond showed higher sealing ability than the ERA Adper Scotchbond 1XT when margins were located on dentin, regardless margin elevation. Gingival margins on enamel together with enamel acid etching provided an excellent sealing with both adhesive systems.

** Key words:**Adhesion, composite inlays, gingival margin, deep margin elevation, marginal seal, nanoleakage test.

## Introduction

Indirect partial posterior restorations constitute an alternative to restore teeth with a severe hard tissue loss (1), which often exhibit subgingival margins. This situation results in biological and operative problems. Biological problems are related with periodontal response to subgingival restorations that may violate biological width and produce gingival inflammation (2). Subgingival margins also hinder impression taking, rubber dam isolation, adhesive procedures, restoration placement and finishing and polishing procedures in cervical area (1). Also, dentin and/or a thin cementum tissue layer are frequently exposed for adhesive procedures instead of enamel limiting a hermetic sealing of this interface and long-term bonding success (1,3-5). In order to solve these circumstances, Dietschi and Spreafico (6) proposed in 1998 the technique “cervical margin relocation (CMR)” that transformed subgingival deep margins in supragingival by the application of a resin composite layer. Other names for this procedure are used such as “deep margin elevation (DME)” (7) and “proximal box elevation (PBE)” (8-10).

Success of indirect partial posterior restorations mainly depends on adequate marginal sealing and adaptation obtained. Adhesive interfaces are exposed to oral conditions, such as masticatory forces, parafunctional habits, temperature fluctuation and chemical substances action that may degrade marginal sealing. Filtration of fluids, bacteria and products through the adhesive interface may cause post-operative sensibility, marginal staining and secondary caries, being the latter, together with tooth or restoration fracture, the main reasons for restoration failure (11,12). Therefore, sealing ability of adhesive materials is of paramount relevance to ensure longevity of these restorations (3), being nanoleakage test one of the laboratory methods proposed for its evaluation and, by extension, the quality of the hybrid layer established (11,13,14).

Up to the present time, there are limited studies evaluating the advantages and limitations of DME technique, and most of them are *in vitro* and focused on marginal adaptation of indirect restorations (8-10,15-18) fracture resistance (10,17) and bond strength (19). Only two studies have evaluated the sealing ability with microleakage tests, reporting a negative effect of DME technique regardless adhesive strategy (18) and resin composite consistency (20). However, currently, there are no available studies that used the nanoleakage test to assess the sealing ability of interfaces and to identify the pattern for interface degradation after DME elevation technique using different adhesive strategies.

Therefore, the objective of the present *in vitro* study was to determine the influence of the gingival margin position and the adhesive strategy used to perform DME in marginal sealing of resin composite inlays by nanoleakage test. The null hypothesis was that similar sealing ability of composite inlays is obtained when gingival margins are located on enamel, dentin or elevated using an etch-and-rinse adhesive (ERA) or a self-etching adhesive (SEA) in combination with a conventional resin cement.

## Material and Methods

Twelve sound third human molars were selected and stored in an aqueous thymol solution at 4ºC until their use for no more than 6 months after extraction.

-Tooth preparation

A mesio-occlusal-distal (MOD) inlay cavity was prepared in each tooth with approximately 10-degree divergent walls using slight taper 80 µm and 25 µm water-cooled diamond burs (Ref 845KR314021 and Ref 845KREF314025, respectively, Komet, Lemgo, Germany) mounted on a high-speed handpiece. Each bur was discarded after five preparations.

All cavities were prepared following the accepted principles for inlay restorations: 2 mm occlusal depth, 3 mm bucco-lingual occlusal width, 4 mm bucco-lingual proximal width, inner angles rounded and no beveled margins.

-Experimental groups 

Teeth were randomly assigned to six experimental groups according to the location of gingival margins of proximal preparations and the adhesive strategy, an ERA or a SEA, used to elevate the subgingival margins and to lute the inlays (Table 1).

**Table 1:**
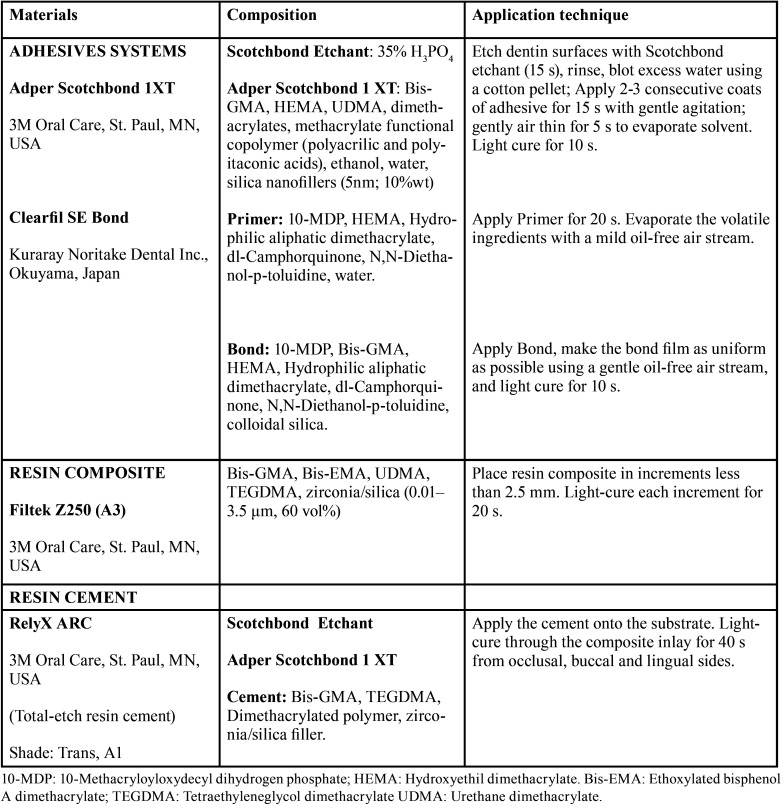
Chemical composition and application technique of the materials tested.

• Enamel + SB1XT: Gingival margins were located on enamel 1 mm above cemento-enamel junction (CEJ) and inlays were luted with the etch-and-rinse adhesive (ERA) system SB1XT (3M Oral Care, St. Paul, MN, USA).

• Dentin + SB1XT: Gingival margins located on dentin 1 mm below CEJ and inlays were luted with SB1XT.

• DME + SB1XT: Gingival margins were prepared on dentin and they were elevated 1 mm above the CEJ by applying a layer of Filtek Z250 (3M Oral Care) with SB1XT. The same adhesive was used to lute the inlays.

• Enamel + CSE: Gingival margins were located on enamel 1 mm above the cementum enamel junction (CEJ) and inlays were luted with the self-etching adhesive (SEA) CSE (Kuraray Noritake Dental Inc., Okuyama, Japan) with selective enamel etching (Scotchbond Etchant, 3M Oral Care).

• Dentin+ CSE: Gingival margins located on dentin 1 mm below CEJ and inlays were luted with CSE.

• DME + CSE: Gingival margins elevated as in DME + SB1XT with CSE adhesive, and it was also used to lute the inlays.

-Inlay fabrication

Impressions of the cavity preparations were taken with high and low viscosity addition polyvinylsiloxane materials (Virtual® Heavy Body and Light Body, Ivoclar Vivadent, Schaan, Liechtenstein) and type 4 stone dies were obtained (KERR-Lab, Orange, CA, USA). Resin composite inlays were fabricated with Gradia Indirect (GC, Tabaskhi-Ku, Tokyo, Japan) applying 2 mm thick layers. Each increment was photocured with the LED unit Elipar S10 (3M Oral Care), for 40 seconds. Inlays were polymerized in an oven (Lumamat 100, Ivoclar Vivadent), using program 3 at 104ºC and high light intensity for 25 minutes.

Inlay inner surface and resin composite used for DME were sandblasted with 50 µm alumina particles (Rondoflex, KaVo, Biberach, Germany), for 10 seconds at a distance of 1 cm. Afterwards, inlays were ultrasonically cleaned in distilled water for 10 minutes and rinsed with alcohol. A layer of SB1XT adhesive was applied on the inlay surface and photopolymerized for 20 seconds.

All inlays were luted using the conventional dual-cure resin composite RelyX ARC (3M Oral Care) following manufacturer´s instructions (Table 1) and under a 1 kg pressure for 5 minutes. The cement was light-cured for 40 seconds from occlusal, buccal and lingual with the same LED unit after excess removal with a brush. Margins were finished and polished with a #11 blade and PoGo polisher points (Dentsply DeTrey, Konstanz, Germany).

-Nanoleakage test 

Teeth restored with the resin composite inlays were stored in distilled water at 37ºC for one week before nanoleakage testing.

All tooth surfaces were covered with two layers of nail varnish 1 mm around the inlay restorations including the elevated margins. Afterwards, molars were immersed in a 50 wt% ammoniacal silver nitrate solution at 37ºC for 24 hours in darkness. Teeth were rinsed with distilled water for 5 minutes and immersed in a photodeveloping solution for 8 hours (Kodak, Rochester, NY, USA) under fluorescent light. Teeth were rinsed with distilled water for 1 minute, and fixed with a 25% glutaraldehyde solution, with a 7.4 pH for 12 hours at 4ºC.

Restored molars were serially sectioned in mesio-distal direction perpendicularly to the gingival wall adhesive interface, and 0.8 thick slices were obtained (Isomet 5000, Buehler, Lake Buff, IL, USA). Smear layer generated was removed with 0.5% phosphoric acid for 60 seconds. Afterwards, specimens were cleaned with 0.2 M sodium cacodylate buffer (pH 7.4) for 1 hour with three changes followed by distilled water for 1 minute.

Afterwards, they were submitted to a dehydration procedure with ascending grades of ethanol according to Perdigao *et al*., 1995 (21). Then, sections were ground with 800 and 1200-grit SiC papers and 1 and 0.3 µm diamond paste (Buehler) using a polishing cloth (Beta Grinder-Polisher, Buehler). They were ultrasonically cleaned in ethanol, air dried and mounted on aluminum stubs. They were sputter coated with 15 nm gold (SCD 005 Sputter Coater, BalTec, Balzers, Liechtenstein) and observed under scanning electron microscopy (Phillips XL30 ESEM, FEI Company, Hillsboro, OR, USA).

Nanoleakage areas were identified as the areas of the interface that displayed silver ions using the scale described by (22) and (23):

• Grade 0: No nanoleakage.

• Grade 1: Nanoleakage up to half the length of the gingival wall.

• Grade 2: Nanoleakage between half of the gingival wall and the axial wall.

• Grade 3: Leakage along the axial wall.

-Statistical analysis 

The results obtained were computed by IBM SPSS 22 (IBM Corporation, Armonk, NY, USA) at alfa = 0.05.

Nanoleakage values obtained for each experimental group were expressed in percentages. The highest value recorded for each proximal box was considered the statistical unit. Results were analyzed by non-parametric Kruskal-Wallis test followed by Mann-Whitney U test for pairwise comparisons with Bonferroni correction.

## Results

No nanoleakage was observed at the interface between the inlays and the conventional resin cement regardless the gingival margin location, nor between the resin composite used for DME and the resin cement. Therefore, the sealing ability was only analyzed at the interface established between enamel or dentin with the resin cement, and between dentin and the resin composite used for DME.

**Figure 1:**
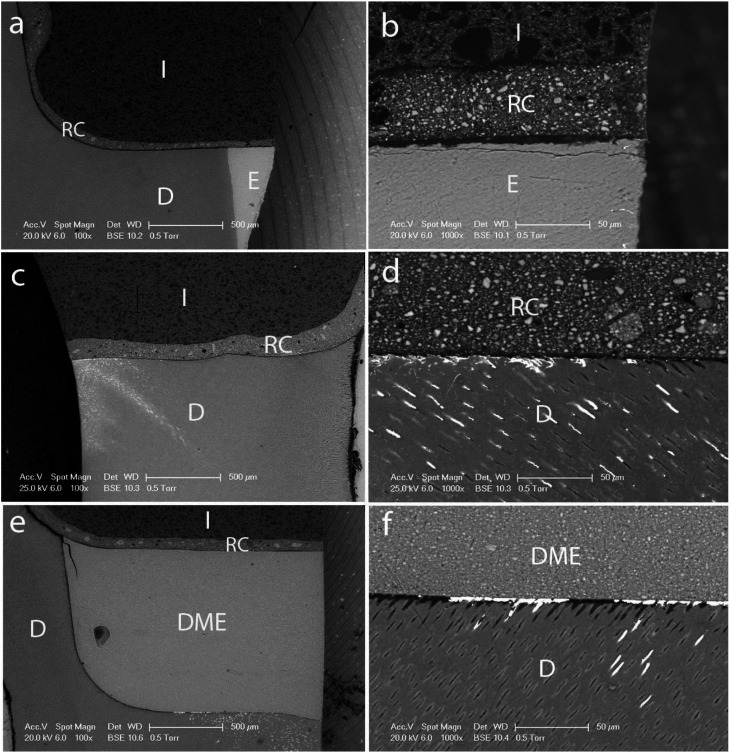
SEM micrographs of nanoleakage determined in the interfaces created after resin composite inlays luting with the ERA system SB1XT and the resin cement RelyX ARC: (a) Enamel + SB1XT 100x and (b) 1000x; (c) Dentin + SB1XT 100x and (d) 1000x; (e) DME + SB1XT 100x and (f) 1000x. E: Enamel; I: Inlay; RC: Resin Cement; D: Dentin; DME: Deep margin elevation.

**Figure 2:**
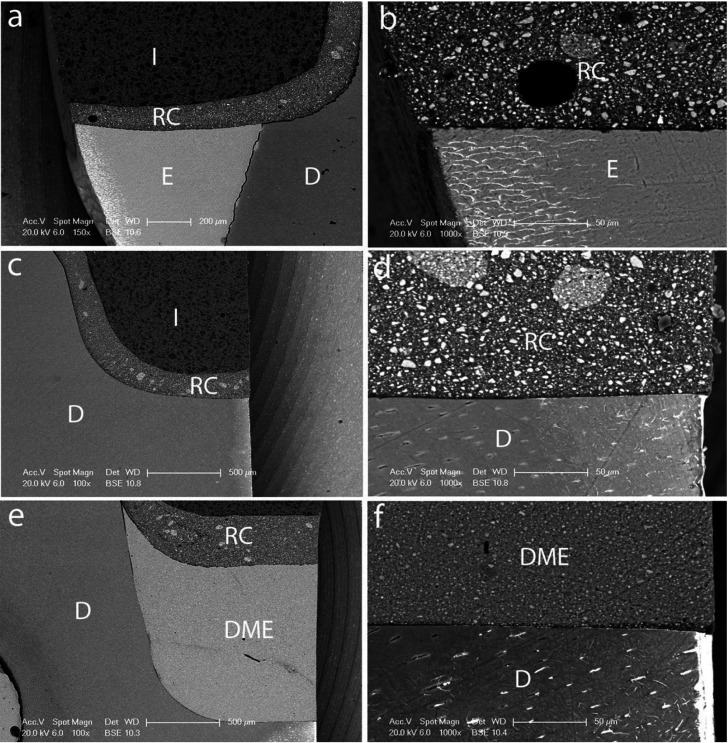
SEM micrographs of nanoleakage determined in the interfaces created after resin composite inlays luting with the SEA system CSE and the resin cement RelyX ARC: Enamel + SB1XT 150x (a) 1000x (b); Dentin + CSE 100x (c) 1000x (d); DME + CSE 100x (e) 1000x (f). E: Enamel; I: Inlay; RC: Resin Cement; D: Dentin; DME: Deep margin elevation.

The nanoleakage values recorded for all experimental groups are shown in Table 2, exhibiting statistically significant differences among them (*p*<0.001). The experimental groups in which gingival margins were located in enamel obtained nanoleakage values of 0, regardless the adhesive system used (Figs. 1a,2a). Statistically similar values were registered for the SEA CSE when gingival margins were in dentin (Fig. 2c) or relocated (Fig. 2e).

**Table 2:**
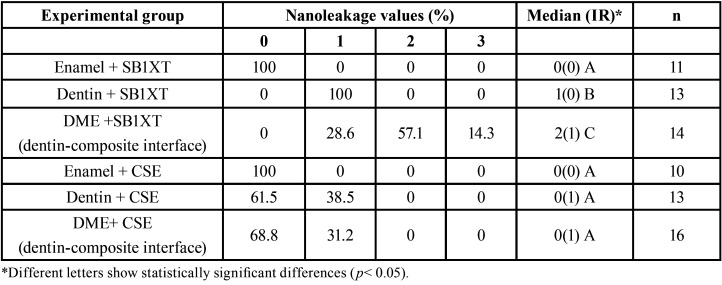
Nanoleakage values determined for in percentages, median (interquartile range, IR) for each experimental group.

For all inlays with gingival proximal margins in dentin and luted after the ERA SB1XT application, silver nitrate deposits were observed penetrating the outer half of the gingival wall (Grade 1) (Fig. 1c). Therefore, sealing ability was intermediary between groups with margins in enamel and those repositioned using this adhesive (Fig. 1e).

The nanoleakage pattern observed was different according to the adhesive system used. In Dentin +SB1XT experimental group, the silver deposits were mainly observed at the bottom of the hybrid layer, spreading into the dentin tubules (Fig. 1d). For DME + SB1XT, silver deposits were also penetrating into the dentin tubules but silver accumulation was detected through the whole adhesive layer (Fig. 1f). For Dentin + CSE and DME + CSE groups, nanoleakage percentages were lower, 38.5% and 31.2%, respectively. In both cases, silver deposits were discontinuous and less concentrated along the adhesive interface, and no ramifications could be detected into the dentin tubules (Figs. 2d,f).

## Discussion

In the present study the influence of the gingival margin location and the adhesive strategy used in marginal sealing was tested. According to our results, the null hypothesis must be rejected as silver nitrate penetration was significantly different if margins were located in enamel, dentin, or elevated when an ERA was used (Table 2).

None of the adhesives hermetically sealed the interfaces created in dentin or when subgingival margins were elevated regardless the adhesive strategy used. These results agree with previous studies that reported high marginal leakage when gingival margins are below the CEJ (24-26). However, higher nanoleakage values were determined for the two-step ERA SB1XT (Fig. 1d,f) in comparison with the two-step SE adhesive CSE (Fig. 2d,f), accordingly to literature that recognizes a better sealing ability in dentin-adhesive interfaces for SEAs (27,28), as well as an influence of the adhesive system used (4,29). This trend was also confirmed in deep proximal margins elevated with an ERA in combination with a flowable composite as they exhibited higher leakage than margins restored with a universal adhesive applied with a bulk-fill flowable composite (18).

The higher silver nitrate penetration recorded for this simplified adhesive, SB1XT, concurs with the reports of other authors (11,30,31). This adhesive contains 5-10 wt% of polyalkenoic acid copolymer in its composition, that is characterized for its high molecular weight that may impair its diffusion through the interfibrillar spaces of the collagen exposed matrix after acid etching of dentin (31). As a result, a non-uniform adhesive-dentin interface is established being more susceptible for hydrolytic degradation (27). Also, over-etching of subgingival dentin when ER adhesives are used has been considered a concern (32).

According to our results, higher sealing ability was determined for SB1XT when inlays were luted to cavity preparations with proximal margins in dentin than when they were elevated with composite (Fig. 1e), in agreement with Köken *et al*. (2018) (20) and Juloski, Köken and Ferrari (2020) (18). It could be that the detrimental effect of polymerization shrinkage on adhesive interfaces is limited when only resin composite cement polymerizes instead of DME technique in which the resin composite layer used to elevate the margin also polymerizes and shrinkages. It should be taken in consideration that polymerization shrinkage stress is related with conFiguration factor being markedly high in the proximal boxes of deep cavities (33).

In contrast, the nanoleakage values obtained for CSE adhesive were significantly lower (Fig. 2c,e), showing a better sealing ability against ammoniacal silver nitrate as previously reported (28). This two-step SEA is considered the “gold standard” for adhesion to dentin (28,34), and several circumstances concur to this excellent adhesive performance. CSE is a mild SEA with a limited demineralizing capacity. Therefore, after its application, hydroxyapatite crystals around collagen matrix are not completely removed (5) and preserved to react with acidic functional monomers. CSE contains 10-MDP as functional monomer that ionically interacts with the calcium present in the hydroxyapatite forming nano-layers of MDP-Ca salts (5,28). The calcium salts formed are sTable and contribute to limit hydrolytic degradation of the adhesive interface and provide a higher long-term durability (35). Moreover, CSE is not a simplified adhesive, and the application of a separate hydrophobic bonding layer is mandatory, preventing water treeing formation, contributing to a higher sealing ability of the adhesive interface (36), and improving hydrolytic degradation resistance and longevity (28).

It should be highlighted that no ammoniacal silver nitrate traces were detected when gingival margins were located in enamel and they were acid etched (Fig. 1a,2a), regardless the adhesive used. This corroborates that acid etching of enamel with phosphoric acid ensures a hermetic marginal seal on which still depends long-term adhesive restorations success (24). This benefit of enamel acid etching is recognized also for mild SEAs, such as CSE, being a selective enamel etching recommended (37). These adhesives, due to their high pH value [1.8] in comparison with phosphoric acid, result in shallower enamel demineralization compared with that of phosphoric acid (38).

Also, the interface between the resin cement and the resin composite inlay, as well as the interface between the resin composite used for DME and the resin cement were free of leakage revealing a perfect seal. Therefore, bonding failures in indirect partial posterior restorations should be only expected from the interface between subgingival margins and the adhesive systems and restorative materials.

One of the limitations of the present study is that sealing ability was evaluated only after one-week water storage. The hydrophilicity of the adhesive interfaces increases with longer aging time producing higher nanoleakage values with different patterns. Therefore, the ability of the interfaces created after DME to avoid leakage should be analyzed after long-term aging by hydrolytic and mechanical degradation to better simulate clinical oral conditions (39). Nevertheless, randomized clinical trials are warranted to confirm the results obtained in the present study and also to evaluate the effects of other factors that may influence the clinical longevity of indirect restorations such as patient´s caries risk, masticatory forces or oral biofilm activity.

## Conclusions

According to the results obtained, the ERA SB1XT showed a lower sealing ability than the SEA CSE when used to perform DME or margins were located in subgingival dentin. The presence of enamel in the gingival margins together with the application of phosphoric acid as the first step of an adhesive procedure using an ERA or a SEA adhesive, achieved a hermetical seal.
